# Enterovirus 68 among Children with Severe Acute Respiratory Infection, the Philippines

**DOI:** 10.3201/eid1708.101328

**Published:** 2011-08

**Authors:** Tadatsugu Imamura, Naoko Fuji, Akira Suzuki, Raita Tamaki, Mariko Saito, Rapunzel Aniceto, Hazel Galang, Lydia Sombrero, Soccoro Lupisan, Hitoshi Oshitani

**Affiliations:** Author affiliations: Tohoku University Graduate School of Medicine, Sendai, Japan (T. Imamura, N. Fuji, A. Suzuki, H. Oshitani);; Tohoku-Research Institute for Tropical Medicine Collaborating Research Center on Emerging and Reemerging Diseases, Muntinlupa City, the Philippines (R. Tamaki, M. Saito, R. Aniceto);; Eastern Visayas Regional Medical Center, Tacloban City, the Philippines (R. Aniceto);; Research Institute for Tropical Medicine, Muntinlupa City (H. Galang, L. Sombrero, S. Lupisan)

**Keywords:** respiratory infections, enterovirus 68, human enterovirus D, pneumonia, children, Philippines, viruses, research

## Abstract

Enterovirus 68 (EV68) is a rare enterovirus associated with respiratory illness that, unlike other enteroviruses, has been identified only from respiratory specimens. We identified EV68 from respiratory specimens of children hospitalized with a diagnosis of severe pneumonia in Leyte, Republic of the Philippines. Twenty-one samples showed high similarity with EV68 by sequencing of 5′ nontranslated region; 17 of these samples were confirmed as EV68 by sequencing of viral protein 1 capsid coding region. Most previously reported EV68 cases had been identified as sporadic cases. All 21 patients we identified had severe illness, and 2 died, possibly the first reported fatal cases associated with EV68 infection. Our study suggests that EV68 may be a possible causative agent of severe respiratory illnesses.

## Abstract 2

TOC summary: Enterovirus 68 was found in 21 children with severe pneumonia.

The genus *Enterovirus* (family *Picornaviridae*) contains 10 species: *Human enterovirus* (HEV) A, HEV-B, HEV-C, HEV-D, *Simian enterovirus A*, *Bovine enterovirus*, *Porcine enterovirus B*, *Human rhinovirus* (HRV) A, HRV-B, and HRV-C. To date, only 3 serotypes have been found for HEV-D: enterovirus 68 (EV68), EV70, and EV94. EV70 is associated with acute hemorrhagic conjunctivitis (*1*), and EV94, a newly found serotype in HEV-D, was identified among enteroviruses associated with acute flaccid paralysis (*2**,**3*). The first EV68 was isolated from hospitalized children with lower respiratory infection in California in 1962 (*4*). Since then, EV68 has been identified sporadically from respiratory specimens (*5**,**6*). EV68 is one of the most rarely isolated enteroviruses; only 26 strains were identified during 36 years of enterovirus surveillance in the United States (*7*). All documented EV68 to date have been reported from the United States and Europe (*3**–**6*); little epidemiologic information is available from Asia and nonindustrialized countries. The clinical significance of EV68 is also not well defined.

Enteroviruses are normally acid resistant and grow at an optimal temperature of ≈37°C, which enables enterovirus to amplify efficiently in the alimentary tract. However, EV68 shares the main characteristics of HRV, which is acid sensitive and grows at a lower optimal temperature (*5**,**8*). These characteristics may explain why EV68 had been isolated only from the respiratory tract (*5*). EV68 and HRV also share high similarity in the 5′ nontranslated region (5′ NTR) (*5**,**8**,**9*). We report a cluster of EV68 infections among hospitalized children with severe acute respiratory illness in the Eastern Viasayas Region of the Philippines during 2008–2009.

## Materials and Methods

### Patients

This retrospective study was conducted at Eastern Visayas Regional Medical Center (EVRMC) in Tacloban City as part of a pediatric pneumonia study. EVRMC is a tertiary government hospital for Eastern Visayas Region, which has a population of ≈3.9 million.

Nasopharyngeal swabs were collected from patients between 7 days and 14 years of age who visited the outpatient clinic at EVRMC and were hospitalized because they met the criteria for a diagnosis of severe pneumonia as defined by the World Health Organization, that is, “a child with cough or difficult breathing and with any of the following signs—any general danger signs (child unable to drink or breastfeed, child is lethargic or unconscious, child vomits everything, or convulsions), chest indrawing or stridor in a calm child—is classified as having severe pneumonia or very severe disease” (*10*). Clinical specimens were collected from 816 children from mid-May 2008 to mid-May 2009. The median age was 9 months; 53% were boys. The study protocol was approved by the institutional review boards of Tohoku University Graduate School of Medicine, Research Institute for Tropical Medicine, and EVRMC. Parents or guardians gave written informed consent for their children to participate in the study.

### Molecular Analysis

RNA was extracted from clinical specimens by using the QIAamp Viral RNA Mini Kit (QIAGEN, Valencia, CA, USA) according to the manufacturer’s instructions. cDNA was synthesized by using random primers (Invitrogen, Carlsbad, CA, USA) and M-MLV Reverse Transcriptase (Invitrogen).

Samples were screened by PCR targeting the 5′ NTR of rhinovirus by using primer pairs DK001 (*11*) and DK004 (*12*) (Table 1). PCR amplicons were purified by using a SUPREC-PCR Kit (TaKaRa Bio Inc., Shiga, Japan) and used as templates in cycle sequencing (ABI Prism BigDye Terminator Cycle Sequencing Ready Reaction Kit, version 1.1; Applied Biosystems, Foster City, CA, USA) in automated sequencers (3130/3130xl Genetic Analyzer, 3730/3130xl DNA Analyzer; Applied Biosystems). For the samples that showed high identity with previously reported EV68 in 5′ NTR sequences, PCR and sequence analysis targeting viral protein (VP) 1 were conducted by using primer pairs 484 and 222 (*5*) and EV68-VP1F and EV68-VP1R (Table 1).

**Table 1:** Primers used for detection and analysis of EV68, the Phillipines*

Primer (reference)	Primer sequence, 5′ → 3′	Location (location no.)†
DK001 (*11*)	CAAGCACTTCTGTTTCCC	5′ NTR (164–168)
DK004 (*12*)	CACGGACACCCAAAGTAGT	5′ NTR (483–501)
484 (*5*)	GGRTCYCAYTACAGGATGT	VP1 (2197–2215)
222 (*5*)	CICCIGGIGGIAYRWACAT	VP1 (2933–2951)
EV68-VP1F	ACCATTTACATGCAGCAGAGG	VP1 (2393–2413)
EV68-VP1R	GACAAGAACTTTTTCAAATGGACAA	VP1 (2683–2707)

*EV, enterovirus; NTR, nontranslated region; VP, viral protein; F, forward; R, reverse. †Location numbers correspond to the genome of EV68 Fermon strain (GenBank accession no. AY426531).

### Sequence Analysis

Sequence analysis was done by using MEGA3.1 software (wwwmegasoftware.net). Phylogenetic trees were generated by using the neighbor-joining method, with maximum-composite likelihood as a substitution model. Similarity was calculated for each genome region by using MEGA3.1 software. Strains of sequences from previous studies that were used for this study are listed in Table 2.

**Table 2:** Sequence data used for analysis of EV isolates, the Philippines*

Strain	GenBank accession no.	Location	Year
EV68.FR37-99	EF107098	France	
EV68.CA62-1	AY426531	United States	1962
EV68.TX99	AY426527	United States	1999
EV68.TX03	AY426526	United States	2003
EV68.NY93	AY426525	United States	1993
EV68.MN98	AY426524	United States	1998
EV68.MD99	AY426523	United States	1999
EV68.MN89	AY426522	United States	1989
EV68.TX02-1	AY426520	United States	2002
EV68.MD02-1	AY426519	United States	2002
EV68.WI00	AY426517	United States	2000
EV68.MO00	AY426516	United States	2000
EV70	DQ201177	Japan	ND
EV94	DQ916376	Egypt	ND
PV1	DQ792910	Greece	ND

*Sequences of 15 referential strains from previous studies, which were used for genetic analysis in this study, are listed above: 12 sequences of EV68, 1 sequence of EV70, EV94 and PV1. EV, enterovirus; ND, no data; PV, polio virus.

## Results

### Sequences of EV68 Strains from the Philippines

Among 816 clinical specimens, a total of 274 were positive by PCR targeting for the 5′ NTR of rhinoviruses, and, of these, 245 were identified as rhinovirus by sequencing of 5′ NTR. Among the remaining 29 specimens, 21 samples had 95.2%–100% similarity to previously reported EV68 by 5′ NTR sequencing. However, sequences of these samples had similarity of <86% with those of EV70 and EV94 (data not shown). Among 8 remaining specimens, 1 specimen was classified as coxsackie virus A16, and other specimens were not identified as any viruses because of poor quality of sequence data. EV68 sequences among the study samples were 96.1%–100% identical to one another (data not shown). These 21 samples were subjected to PCR for the VP1 region. The VP1 region was amplified only for 17 of 21 positive samples. The similarity that was calculated for the VP1 region was compared with the sequences of EV68 from the Philippines, EV68 strains from other countries, EV70, and EV94 (Table 3). VP1 sequences from the Philippines had similarity of 86.2%–95.3% with those of the strains from other countries and had similarity of 90.6%–100% to the viruses in the Philippines, while they had similarity of <65% with EV70 and EV94.

**Table 3:** Percentage similarity among viral protein 1 sequences of EV68 from the Philippines and reference strains of EV68, EV70, and EV94 from other countries*

Strain (reference)	FR37-99 (*3*)	TX03 (*5*)	NY93 (*5*)	CA62-1 (*5*)	Ph343	Ph451	Ph513	Ph561	Ph575	Ph43	EV70 (*12*)	EV94 (*12*)
FR37-99 (*3*)		92.8	90.7	88.1	93.2	93.2	93.2	89.4	94.1	94.1	62.3	67.8
TX03 (*5*)			93.6	89.0	92.8	92.8	92.8	94.1	93.6	93.6	63.6	67.8
NY93 (*5*)				90.3	92.4	92.4	92.4	90.3	93.2	93.2	64.8	66.9
CA62-1 (*5*)					89.8	91.9	91.9	88.1	91.9	91.9	64.4	66.1
Ph343						97.5	97.5	91.1	98.3	98.3	62.3	66.1
Ph451							100	91.9	99.2	99.2	63.1	66.1
Ph513								91.9	99.2	99.2	63.1	66.1
Ph561									92.8	92.8	63.6	65.7
Ph575										100	63.1	66.5
Ph43											63.1	66.5


*In the table, homology of viral protein 1 sequences between the strains are listed horizontally, and those listed vertically are indicated in the box where the rows for each strain meet. EV, enterovirus.

The sequence data described in this paper have been deposited in the GenBank sequence database under accession nos. AB569257–AB56924. Because of high similarity of VP1 sequences among the analyzed samples, RNA extraction, PCR, and sequencing for VP1 were repeated for selected samples to exclude a possibility of contamination. All retested samples showed identical results.

**Figure 1:**
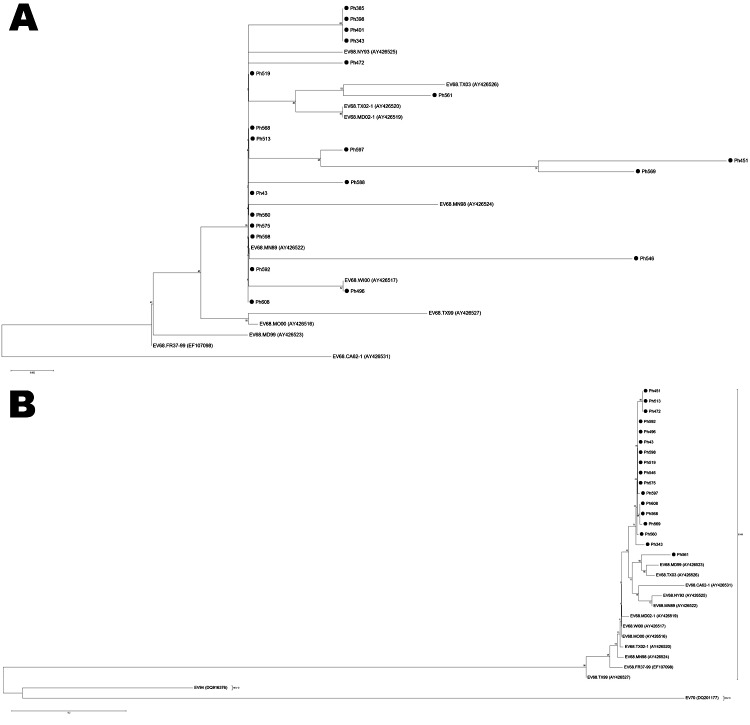
Phylogenetic trees of selected enterovirus (EV) 68 strains, based on the nucleotide sequence of 2 genomic regions: A) partial 5′ nontranslated region and B) partial viral protein 1. EV68 strains analyzed in this study are indicated by black circles. Phylogenetic analysis was performed by using nucleotide alignments and the neighbor-joining method, as implemented in MEGA software (www.megasoftware.net). Poliovirus 1, EV70, and EV94 sequences were used as outgroups. Scale bar indicates number of nucleotide substitutions per site.

Phylogenetic trees were based on the 5′ NTR and VP1 gene sequences, including the sequences of previously reported EV68. On the phylogenetic tree based on VP1 sequences, sequences from the Philippines fit into the EV68 cluster in which all EV68 stains from other countries are located. This cluster is clearly distinguishable from clusters of other enteroviruses, including EV70 and EV94 (Figure 1, panel B). Among 17 EV68 strains from the Philippines, no significant variation of VP1 sequences was observed, except for Ph561, which was not grouped together with strains from the Philippines but was grouped with EV68.TX03, which was identified in Texas (United States) in 2003, and EV68.MD99, which was identified in Maryland (United States) in 1999 (Figure 1, panel B). On the phylogenetic tree based on 5′ NTR sequences, 21 sequences from the Philippines fit into the EV68 cluster together with other EV68. There were variations among viruses from the Philippines in 5′ NTR, especially Ph451 and Ph569, which formed a separate branch from other EV68 viruses. Ph561 appears to be closely related to TX03. This sample was also grouped into the same distinct lineage with TX03 on the phylogenetic tree based on 5′ NTR (Figure 1, panel A). Moreover, Ph561 had 94.9% similarity to TX03, while it was <90.9% identical to EV68 strains from other countries.

Within the enterovirus species, serotype classification is based on nucleotide similarity in the VP1 region (*13**–**15*). It was proposed that they should be classified into the same serotype if they have >75% nucleotide similarity in the VP1 region (>85% amino acid similarity) (*13**,**15*). Sequence analysis of VP1 revealed that EV68 detected in the study had similarity of >86.2% with previously identified EV68, which matched the proposed criteria. Phylogenetic tree of VP1 sequences also confirmed that EV68 detected in the study were located among EV68 clusters with other EV68 strains reported in previous studies.

VP1 sequences were not obtained for 4 specimens among 21 that were positive for the 5′ NTR region, probably due to the low virus RNA content in the samples. Reports have shown that sequencing of the 5′ NTR is not reliable for serotype classification due to high frequency of recombination in this region (*16**–**18*). However, similarity and phylogenetic analysis of the 5′ NTR indicated that all 21 specimens had high sequence similarity with previously identified EV68. These facts indicate that all 21 patients had EV68 infection.

On the phylogenetic tree based on VP1 sequences, only Ph561 did not cluster with other strains from the Philippines, but instead clustered with strains from the United States (TX03 and MD99). Ph561 was <91.9% identical to other strains from the Philippines, while other strains from the Philippines were >97.6% identical to one another. This finding suggests that the particular virus had a different origin from others and that >2 genetically different EV68 with divergent VP1 sequences were circulating. However, there was no unique geographic or temporal characteristic of Ph561, because this virus was identified from the patient from Tacloban City in December 2008.

### Descriptive Epidemiology of EV68

EV68 was detected in 21 of 816 samples by molecular methods. These samples represent 2.6% of 816 samples collected in this study.

**Figure 2:**
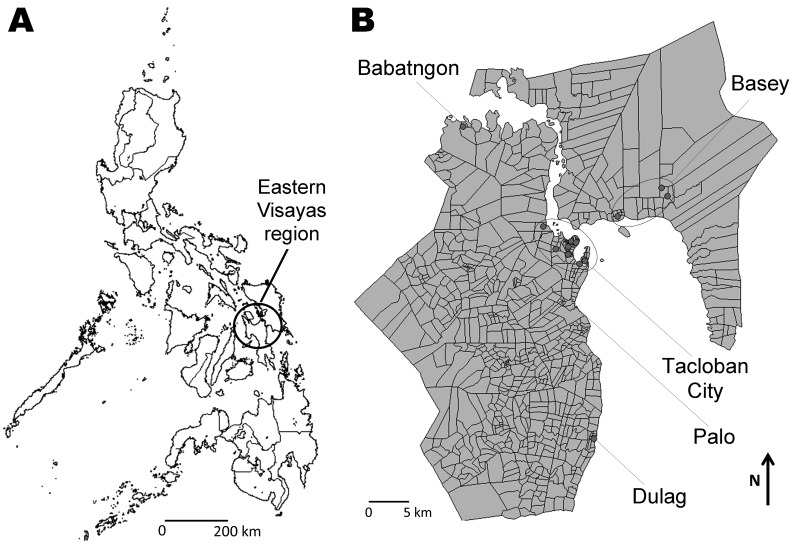
Geographic distribution of residences of patients in whom enterovirus 68 was detected in the Philippines, May 2008–May 2009. A) Eastern Visayas Region in the Philippines; B) expanded Eastern Visayas Region. Address information was obtained from parents of the children. Locations for 6 patients were unknown.

**Figure 3:**
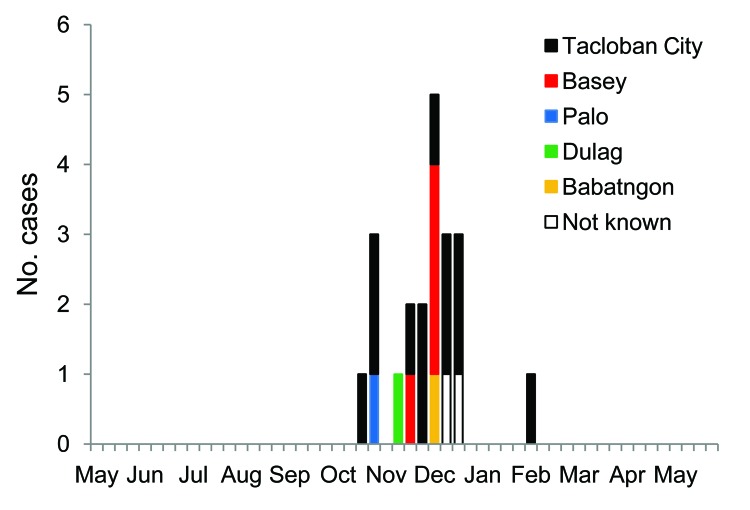
Temporal and geographic distribution of enterovirus (EV) 68 cases in Eastern Visayas Region in the Philippines, May 2008–May 2009. Address information was obtained from parents of the pediatric patients. The graph shows the number of reported EV68 cases of each week in Eastern Visayas Region, and a report from a different city in the region is indicated with a bar of different color. The weeks with no bars indicate no reported cases of EV68.

Geographic distribution of the patients who had positive EV68 samples is shown in Figure 2. Among 21 patients found to have EV68 positive specimens, 12 were identified in Tacloban City, and the 7 remaining were from surrounding areas (Figure 2). Patients with EV68 infection sought treatment beginning in the third week of October 2008, the number of patients peaked in the 1st week of December, and EV68-positive cases were found after March 2009 (Figure 3).

The patients in whom EV68 was detected were from 1 month to 9 years in age; median age was 21 months. Eight of 21 patients were girls, and 13 were boys. Common signs and symptoms the patients had included cough (100%), difficulty in breathing (85.7%), wheeze (66.7%), and chest in-drawing (100%). Fifteen patients were discharged, but 2 patients died during hospitalization. The outcome of 4 patients was not obtained. The 2 deaths represented 9.5% of 21 patients in whom EV68 was detected, while the rate of deaths associated with HRV infection in the study period was 6.1%. Among 816 patients with severe pneumonia, 70 died in the study period. The 2 patients who died with EV68 infection represented 2.9% (2/70) of the total deaths of patients with pneumonia.

## Discussion

We reported a cluster of EV68 infections among hospitalized children with a diagnosis of severe pneumonia in Leyte province, the Philippines. EV68 was identified in 21 cases by PCR and sequencing between October 2008 and February 2009. The number of reported cases of EV68 is limited, and most cases have been reported as sporadic cases (*4**–**6*). As far as we know, clusters of EV68 reported to date include only 2 reports: 4 cases among hospitalized children with lower respiratory tract infections in California in 1962 (*5*) and 7 cases with febrile respiratory illness among military recruits in San Diego during 2004 and 2005 (*6*). We report a large cluster of EV68 that includes fatalities.

Limitations of this study include the fact that we only tested samples from hospitalized patients with severe cases of pneumonia. There might have been many more cases among patients with milder illness in the community. Most of the cases were identified in patients from Tacloban City, the biggest city in the region, but additional cases were also identified from neighboring communities. It indicates that the virus was circulating in a relatively large geographic area during an extended period of 5 months. It is possible that a rare outbreak of EV68 happened to be detected during this study period. It is also possible that EV68 is endemic and causing annual or cyclic outbreaks in this area. Further studies are necessary to define epidemiology of EV68 in the Philippines.

EV68 shares several phenotypic characterizations with rhinovirus, including acid lability and a lower optimal growth temperature (*5**,**8*). The virus that was previously classified as HRV87 was shown to be identical to EV68 (*8**,**9*). It was also shown that EV68, like other HRV, replicates well in the bronchial epithelial cells (*5*). Human enteroviruses are commonly isolated from stool specimens; however, all previously identified EV68 had been isolated from respiratory specimens (*4**,**6**,**13*). Clinical spectrum of EV68 infection is still not well defined. However, EV68 may have similar clinical illness with HRV. HRV was thought to only cause mild upper respiratory infection. Recently it has been shown that HRV is commonly associated with lower respiratory infection (*19*) and exacerbations of asthma (*20**–**23*). The first isolates of EV68 were detected in hospitalized children with lower respiratory infection (*4*). In the present study, all EV68-positive cases were in hospitalized children with a diagnosis of severe pneumonia, and 2 children died. Acute respiratory infection, particularly pneumonia, is still a major cause of child deaths in nonindustrialized countries (*24**,**25*). The clinical importance of EV68, including its etiologic role in severe respiratory infection, should be further defined.

The sequences of 5′ NTR are similar between HRV and EV68 (*8*). Therefore, EV68 was detected by reverse transcription PCR (RT-PCR) targeting 5′ NTR of HRV (*6*). However, the sensitivity of EV68 detection by RT-PCR by using primers for 5′ NTR of HRV has not been validated. In our study, samples were screened by RT-PCR using primers for 5′ NTR of HRV, which may have missed some EV68 positive cases.

In conclusion, our study highlighted the potential importance of EV68 as a causative agent of severe respiratory infection, which is a leading cause of pediatric deaths in nonindustrialized countries. Clinical and public health impact of EV68 may be underestimated because isolation of EV68 is relatively difficult and requires the use of fibroblast cells. Sporadic cases of EV68 have been detected by virologic surveillance, which suggests that EV68 is circulating in the community. A careful laboratory testing approach may be able to detect more EV68 among patients with respiratory infections.
